# Mechanical and environmental performance of sugarcane Bagasse Ash from Khyber Pakhtunkhwa in sustainable concrete

**DOI:** 10.1038/s41598-025-10383-6

**Published:** 2025-07-08

**Authors:** Muhammad Fahad Ullah, Hesheng Tang, Arshad Ullah, Zsolt Toth, Mahmood Ahmad, Abdullah Alzlfawi

**Affiliations:** 1 Department of Disaster Mitigation for Structures, College of Civil Engineering, Tongji University, Shanghai, 200092 China; 2 Department of Geotechnical Engineering, College of Civil Engineering, Tongji University, Shanghai, China; 3 Department of Transportation and Geotechnical Engineering, National University of Sciences and Technology, Islamabad, Pakistan; 4 Faculty of Wood Engineering and Creative Industries, University of Sopron, Sopron, Hungary; 5 Department of Civil Engineering, University of Engineering and Technology, Peshawar (Bannu Campus), Bannu, 28100 Pakistan; 6 Department of Civil and Environmental Engineering, Majmaah University, Al Majmaah, Saudi Arabia

**Keywords:** Sugarcane bagasse ash, Alkali-silica reactivity, CO_2_ emissions, Mechanical strength, Cost savings, Engineering, Civil engineering, Structural materials

## Abstract

In recent decades, the partial substitution of cement with sugarcane bagasse ash (SCBA) has received attention for construction applications because of its pozzolanic characteristics. However, regional-scale studies are encouraged to increase the use of SCBA at the industrial level. Limited literature is available on the effect of SCBA on concrete Alkali-silica reactivity (ASR), CO_2_ emissions and economic feasibility. In the current study, the influence of adding 0%, 5%, 10%, and 15% locally available SCBA from Khyber Pakhtunkhwa on the consistency, mechanical strength, ASR, N_2_ adsorption, mineralogy, microstructure, and elemental compositions of concrete was investigated. In addition, CO_2_ emissions and cost analysis were conducted for all the concrete mixes. Experimental findings revealed that consistency increased with the addition of SCBA percentages, whereas a delay in the setting time was recorded. The Compressive strength (CS) and split tensile strength for all SCBA-based mixtures increased with ageing, due to the finer particles and higher surface area of SCBA. Additionally, SCBA effectively reduces the expansion resulting from the alkali-silica reaction. The incorporation of SCBA significantly improved the microstructure with no sign of cracks, resulting in higher reactivity and the formation of additional CSH gel than the control mix. The findings confirmed that incorporating 10% of SCBA resulted in eco-friendly construction material with enhanced strength and cost savings. Furthermore, this study is beneficial to promote the use of locally available SCBA in concrete instead of disposal in landfills.

##  Introduction

Ordinary Portland cement (OPC) concrete is the most frequently human-made commodity on the earth^1^. The annual production of concrete is around 4.4 billion tons worldwide, and the demand is expected to reach 5.5 billion tons by 2050 due to rapid urbanization in developing countries^2^. The production of one ton of OPC requires 110 kWH and releases almost 7% of worldwide CO_2_ emissions^3,4^. Therefore, partial cement replacement with environmentally friendly materials known as supplementary cementitious materials (SCMs) has proven to be a successful strategy for mitigating the environmental impact of concrete^5^. The SCMs not only produce environmentally sustainable concrete but also produce blends with higher workability, strength, and durability^1,6^. In the past few decades, SCMs obtained from industrial waste have been utilized as a partial substitute for OPC. The most commonly used SCMs include Granulated blast furnace slag^7,8^ rice husk ash^5,9^ fly ash^10,11^ and silica fume^7,12^. Concrete’s mechanical properties were significantly improved by adding these pozzolanic materials^10,13^.

The cement industry would need 1.58 billion tons of SCMs per year for almost 50% of the clinker factor of OPC should be replaced with eco-friendly materials to reduce 1 billion tons of CO_2_ annually^1^. Rice husk ash, granulated blast furnace slag, and fly ash are the most significant among the SCMs because they have been already utilized in significant proportion by the cement construction industry^14,15^. Unfortunately, the production of these byproducts is anticipated to reduce in the coming years^16^. In addition, the demand for cement continues to grow and rapidly transform the industry to increase exports/imports of SCMs between countries^14^which has led to seeking new sources of SCMs. One possibility is the use of biomass ashes as a partial replacement for OPC in concrete such as RHA^7,17^SCBA^18–20^and wheat straw ash^21,22^. Vegetable ashes significantly reduce the permeability, resulting in improved mechanical strength of concrete^23,24^. SCBA and RHA are the most frequently used among vegetable ashes^16^. Sugarcane contains 25-30% of the sugarcane bagasse^25^. The SCBA is generated during the combustion process to produce thermal energy, which is accumulated in landfills^26^.

**Fig. 1:**
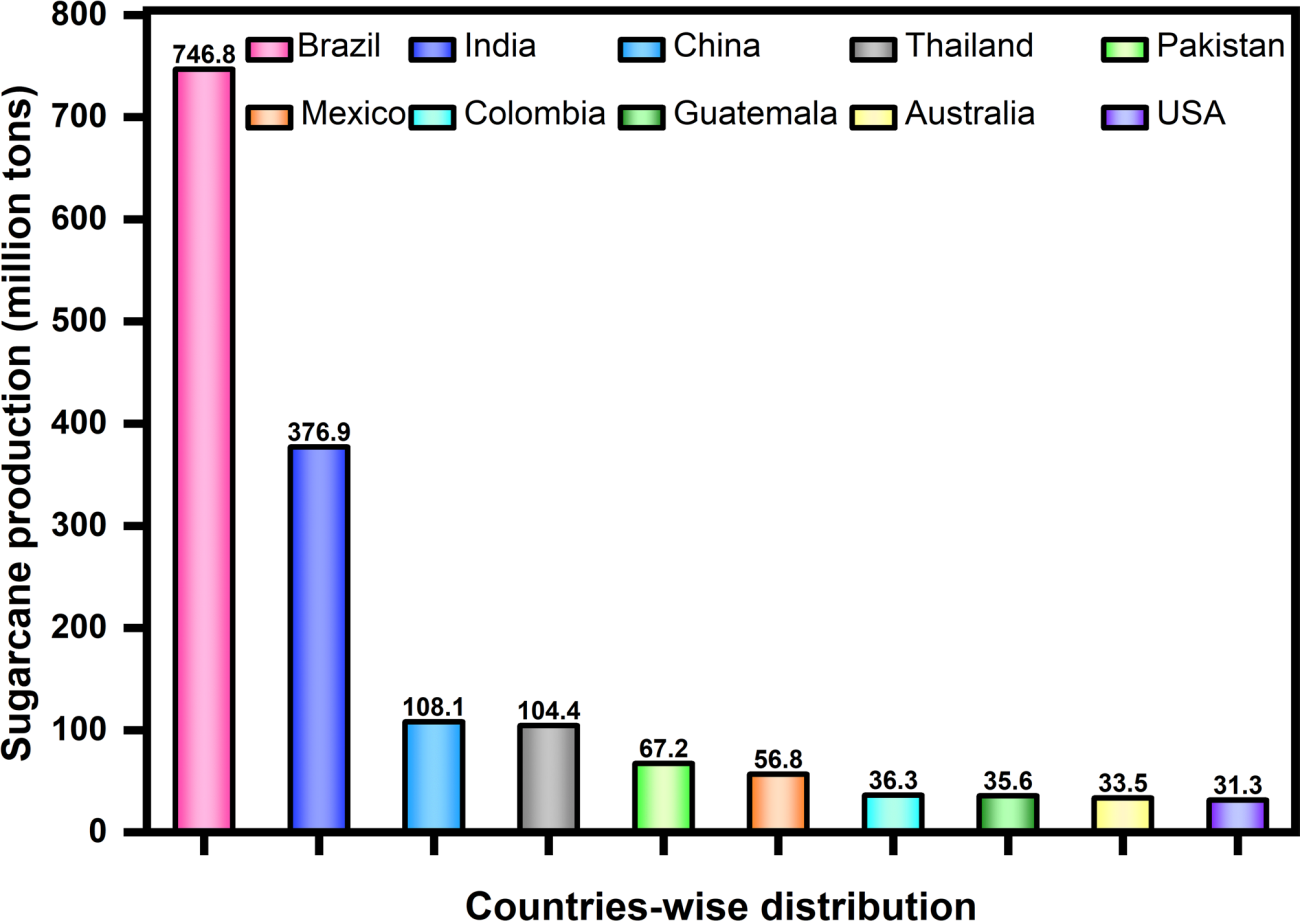
Global production record of the 10 countries with the largest sugarcane production^27^.

The global sugarcane production recorded in 2018 was around 1.9 billion tons. The highest sugarcane crop production countries in 2018 are demonstrated in Fig. 1, which contributes to 80% of the total worldwide sugarcane production^27^. Pakistan is the sixth-highest sugarcane-producing country contributing to 67.2 million tons of sugarcane in 2018. Recently, according to the Pakistan Economic Survey 2023-24, sugarcane production in Pakistan increased to 87.638 million tons^28^. Considering that 6.6 kg of ash is produced from one ton of sugarcane^15^. It can be evaluated that around 0.5784 million tons of SCBA were produced in Pakistan for the year 2023–2024. The SCBA has not been utilized effectively and disposed of locally in landfills^25^which has not only taken valuable lands but is also believed to contaminate the groundwater^27^. Hence, the utilization of SCBA as SCM in concrete not only decreases the carbon footprint but also decreases the environmental impacts due to the SCBA stockpiled in landfills.

Several researchers have highlighted efforts to explore the potential utilization of SCBA as SCM because of its higher pozzolanic properties and availability in large proportions. Literature showed that SCBA can be utilized as a partial substitute for OPC by up to 30%^25^^29^, and 40% of the binder weight in concrete^30^. Ganesan, et al.^31^ reported that the optimal dosage or percentage of SCBA as an OPC replacement can be 20%, leading to high early mechanical strength, reduced permeability, and significant resistance to chloride permeation. The SCBA addition of up to 15% tends to reduce the porosity and enhance the CS, however, the specimen containing SCBA up to 10% showed a lifespan exceeding 50 years in all environments, while those with 15% SCBA specimen had a lifespan of almost 26 years in the industrial environment. Previous studies by Quedou, et al.^32^ and Krishna, et al.^33^ recommended 10% of SCBA as the optimal replacement for the OPC to achieve enhanced properties such as higher CS, flexural strength, and splitting tensile strength. SCBA’s fine particles (< 10 μm) and high silica content have been shown to reduce chloride penetration by up to 45% compared to OPC, primarily through improvement in pore structure and chloride-binding capacity^34^. Durability improvements in SCBA-modified concrete arise from decreased permeability and conductivity, attributable to microstructural densification that effectively restricts chloride ion diffusion^35^. Concrete with 10% SCBA replacement in lightweight foam concrete demonstrates a reduction in water absorption, primarily due to microstructural refinement from pozzolanic reactions that decrease capillary porosity and interfacial transition zone (ITZ) permeability, validated by reduced chloride diffusion and sorptivity^36^. SCBA mixtures may initially exhibit higher carbonation depths due to consumption of portlandite, but the resistance is improved in the long term (90 days) compared to the control mix due to densification of the matrix. The best performance is achieved with 10–15% replacement, where the pore refined structure maintains alkalinity^37^.

As highlighted by Li, et al.^27^ further investigations are needed on the SCBA-based concrete long-term behavior, cost saving, environmental effect, and alkali-silica and abrasion resistance. In addition, the lack of understanding of the availability and geographical distribution of SCBA is identified as the main limitation of their industrial-scale use. Regional-scale surveys and research are essential to comprehensively investigate SCBA properties and increase their potential usage^38^. Therefore, a study is direly needed to investigate the properties of locally available SCBA in concrete as well as to evaluate their environmental effect and cost saving, which will help to encourage the usage of SCBA on a regional scale. Several key challenges must be addressed to promote regional adoption of SCBA as a sustainable cement replacement. These include ensuring quality consistency, durability, availability, cost-effectiveness, and increasing stakeholder awareness. By implementing standardized quality control, regional research, durability testing, supply chain optimization, and market incentives, SCBA can become a viable alternative to traditional cement. These efforts will not only enhance SCBA-based concrete performance but also facilitate large-scale adoption, advancing sustainable construction practices.

The purpose of this study was to assess the potential of locally available SCBA for OPC replacement to promote sustainability in concrete production, reduce the disposal of SCBA in landfills, and enhance regional understanding of SCBA for industrial use. SCBA was collected from various districts of Khyber Pakhtunkhwa based on production and incinerated under controlled environmental conditions to obtain the maximum amount of silica and analyzed using XRD (X-ray Diffraction) and XRF (X-ray Fluorescence). Cement matrices with different percentages of SCBA (0%, 5%, 10%, 15%) were prepared, and their consistency, setting times, compressive strength, splitting tensile strength, and ASR were evaluated. In addition, XRD, SEM-EDX, and N_2_ Adsorption analyses were conducted to determine the mineralogy, pore and microstructural, elemental compositions, and behavior of SCBA-based paste. Furthermore, the carbon emissions and cost analysis were evaluated for the potential use of SCBA in the concrete industry.

## Materials and methods

### Materials

#### Cement

According to ASTM C-150^39^, Ordinary Portland Cement (Type-I), locally available and manufactured by the Bestway Cement factory in Pakistan, was utilized to prepare the concrete samples in this research work. The different physical properties and oxide composition of the OPC were determined in the laboratory. The specific gravity and Blaine air fineness of the OPC were 3.15 and 3300 cm^2^/gm. The chemical compositions of the OPC are listed in Table 1.

**Table 1:** Chemical compositions of OPC.

Chemical compositions	Percentage
SiO_2_	21
CaO	61.7
Al_2_O_3_	5.04
Fe_2_O_3_	3.24
MgO	2.56
SO_3_	1.51
K_2_O	0.62
Na_2_O	0.13
LOI	1.83
IR	0.54
Free lime	0.68

#### Bagasse Ash

##### Collection of sugarcane Bagasse

Sugarcane Bagasse Ash (SCBA) chemical compositions highly depends on its source, organic composition, and incineration method. As a result of the unique meteorological and geographical conditions of the region, various sources of SCBA have different chemical compositions. Researchers noticed that the chemical compositions of SCBAs vary and depend on the meteorological conditions as well as the geographical locations of the Sugarcane Bagasse. Sugarcane Bagasse specimens were collected from three different regions in the Khyber Pakhtunkhwa Province such as Malakand, Charsadda, and Mardan regions based on climatic conditions and geographical variations. The Khyber Pakhtunkhwa Bureau of Statistics report “Development Statistics of Khyber Pakhtunkhwa 2023” lists the net sugarcane production in the selected locations for sampling shown in Table2^40^.

**Table 2:** Sugarcane production in Khyber Pakhtunkhwa from 2019–2022, data taken from^40^.

Locations	2019–2020		2020–2021		2021–2022	
	Area (Hectare)	Production (Tons)	Area (Hectare)	Production (tones)	Area (Hectare)	Production (tons)
KPK	109,359	5,753,975	107,438	5,627,545	95,098	4,909,950
Malakand	4,860	192,408	4,880	195,307	4,890	196,804
Charsadda	29,655	1,735,064	31,148	1,806,142	28,462	1,640,630
Mardan	30,172	1,298,603	29,827	1,277,839	28,935	1,216,537

##### Soil properties

Soil plays a pivotal role in plant and crop growth and productivity. Soil is influential in producing healthy plants and crop proper growth. Several significant elements and their oxides are found in soil, such as silicon (Si), calcium (Ca), manganese (Mn), iron (Fe), magnesium (Mg), aluminum (Al), potassium (K), sodium (Na), and phosphorus (P) separated from parent source generally covered by igneous or sedimentary rocks which greatly influenced the production of crops. In addition, silica is identified as a critical part of the soil profile and a fundamental element of plant cell walls, which influences the physical properties and oxide composition of soil and crops. Additionally, alumina contributes to physiochemical soil properties, and iron oxide contributes to soil fertility; additionally, other oxides Na_2_O, MgO, and K_2_O are also present in trace amounts affecting soil properties^5^. In the initial phase, Bagasse samples were collected from various regions based on soil properties having the maximum amount of Silica as major oxide; the types and major oxide compositions for the selected regions are illustrated in Table 3.

**Table 3:** Soil types and oxide compositions of the selected regions of Khyber Pakhtunkhwa^5^.

Location	Soil Type	Major Oxides (Wt.%)		
		SiO_2_	Al_2_O_3_	Fe_2_O_3_
Malakand	Loamy, Shallow	45–57	10–21	2.9–4.5
Charsadda	Loam	50–72	13–19	2.74–6.1
Mardan	Loam	48–57	09–12	3.1–5.89

##### Production of SCBA

Sugarcane Bagasse Ash (SCBA) was combusted to evaluate maximum SiO_2_ at the Pakistan Council of Scientific and Industrial Research (PCSIR), Peshawar. Upon incineration, the changes in the chemical characteristics of the SCBA depend on the degree of applied temperature and different time duration. The sugarcane bagasse obtained from selected areas of KPK was burned under a controlled temperature of 600–700 °C using a Ferro cement drum kept for 24 h. After cooling at an ambient temperature, the SCBA was grounded in a rotary mill at 15 rpm for 12 h to obtain finer SCBA. The SCBA was analyzed for the chemical compositions and mineralogy using X-ray fluorescence (EDX-7000, Shimadzu, Japan) and X-ray diffraction (JDX 3532, JEOL, Japan) installed in Central Resource Labs (CRL), University of Peshawar.

X-ray fluorescence (XRF) analysis was conducted in order to evaluate the oxide compositions of SCBA. Table 4 demonstrates the findings of XRF analysis of SCBA samples collected at three locations, Malakand, Charsadda, and Mardan of Khyber Pakhtunkhwa, Pakistan. The main chemical compositions found in the XRF analysis are SiO_2_, Al_2_O_3_, CaO, K_2_O, Fe_2_O_3_, MnO, and traces of other oxides. The results showed that all three samples mainly contain SiO_2_ in their chemical composition and are thus beneficial in concrete as a partial substitute binder material. The physical and mineralogical characteristics of SCBA play a crucial role in governing its pozzolanic activity and thus were thoroughly analyzed. The SCBA samples exhibited a Blaine fineness of 4720 cm²/g, indicating a highly fine material that promotes enhanced pozzolanic reactions due to increased surface area available for reaction with calcium hydroxide. The relatively high specific surface area is beneficial for densifying the concrete microstructure^35,41^. The measured LOI values ranged between 1.435% and 7.314%, with Charsadda SCBA having the lowest LOI, suggesting lower carbon residues and a higher degree of combustion completeness. A low LOI typically correlates with better pozzolanic activity because unburnt carbon can inhibit cement hydration^42^.

The analysis of the chemical composition indicates that the cumulative percentage of SiO_2_, Al_2_O_3_, and Fe_2_O_3_ exceeds 70% across all evaluated sources (Malakand: 78.26%, Charsadda: 79.01%, Mardan: 75.32%). This characteristic aligns with the established criteria for classifying a material as pozzolanic, as outlined by ASTM standards C618^43^. The high SiO_2_ content (over 60%) also suggests the potential for strong pozzolanic reactivity. The elevated SiO_2_ content combined with the fine particle size strongly indicates the presence of reactive silica phases typically found in SCBA. These characteristics support the positive impact of SCBA on the mechanical strength and durability performance of concrete, as observed in this study.

**Table 4:** Oxide compositions of SCBA collected from different districts of Khyber Pakhtunkhwa.

Oxide compositions	Percentage distribution		
	Malakand	Charsadda	Mardan
SiO_2_	61.548	62.839	60.018
Al_2_O_3_	10.127	10.878	10.686
CaO	9.631	8.913	9.351
K_2_O	6.828	6.581	7.554
Fe_2_O_3_	4.590	5.293	4.626
MnO	1.247	3.295	0.323
P_2_O_5_	0.668	0.795	0.072
SO3	0.061	0.720	0.061
TiO_2_	0.028	0.530	0.014
SrO	0.011	0.042	0.016
CuO	0.009	0.023	0.007
ZrO_2_	0.003	0.017	0.003
Rb_2_O	0.002	0.016	0.002
LOI	5.247	1.435	7.314
SiO_2_ + Al_2_O_3_ + Fe_2_O_3_	78.265	79.015	75.322

The XRF analysis showed that SCBA samples collected from Charsadda have a maximum percentage of SiO_2_, thus the XRD analysis was conducted for the mentioned SCBA sample only. SCBA is primarily composed of silica (SiO_2_), alumina (Al_2_O_3_), iron oxide (Fe_2_O_3_), and varying amounts of calcium oxide (CaO)^34^. The silica content plays a significant role in pozzolanic reactivity, with higher silica content resulting in improved strength and durability^44^. The chemical composition of SCBA exhibits considerable variability, which is influenced by several factors, including combustion temperature, the method of burning, and the specific soil type in which the bagasse is cultivated. These factors influence the amorphousness of silica, calcium content, and the presence of alkalis, all of which affect the pozzolanic reactivity and overall performance of concrete^37^. The presence of high unburnt carbon content in SCBA results in reduced pozzolanic reactivity. This phenomenon can be attributed to the predominance of less reactive fibrous particles within the ash, which adversely affects its overall reactivity in cementitious applications^37,45^. The variability in SCBA composition should be carefully considered when using it as a supplementary cementitious material to ensure optimal concrete properties.

**Fig. 2:**
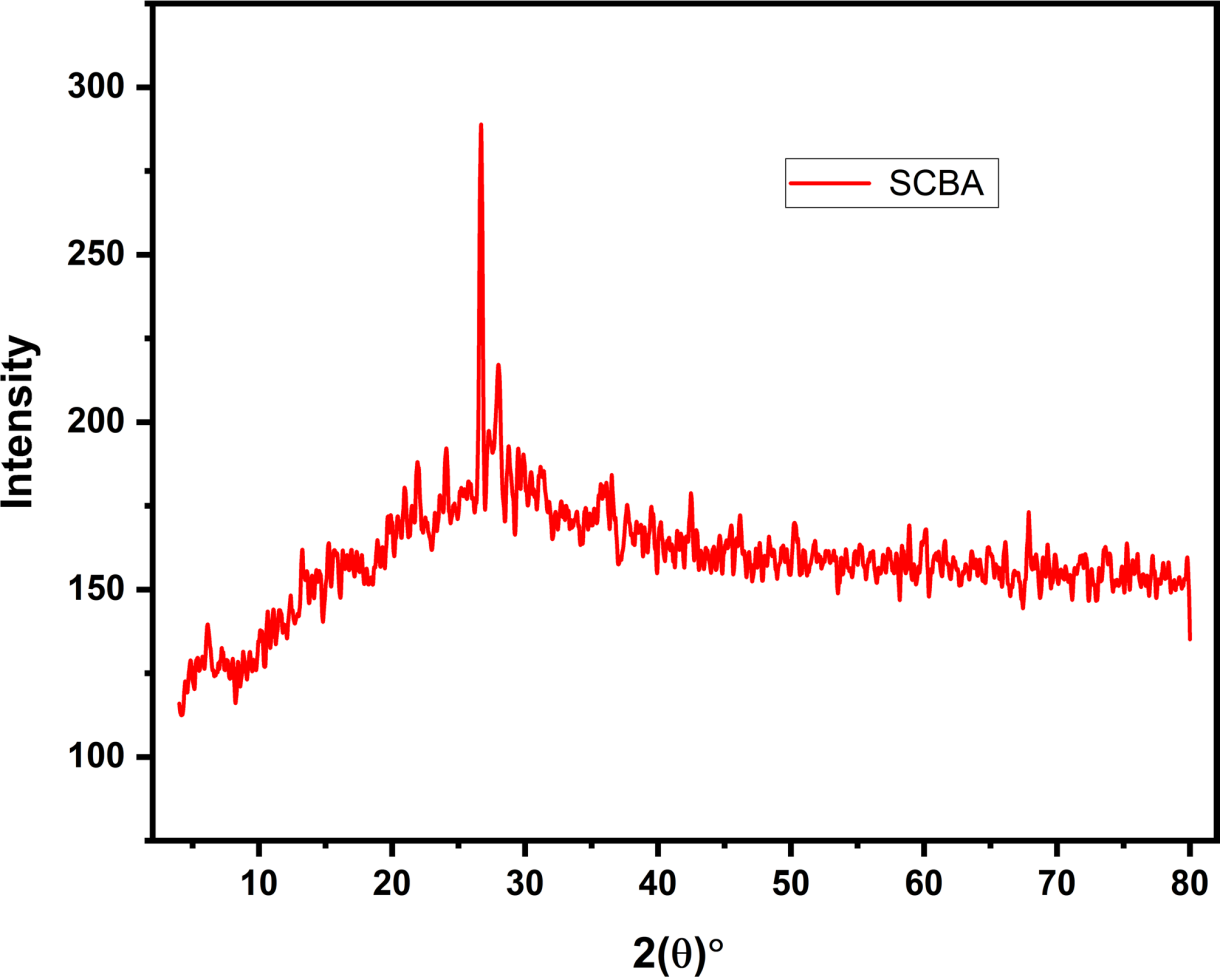
XRD pattern of Charsadda SCBA obtained after the combustion method.

The XRD results of the SCBA collected from the Charsadda site are displayed in Fig. 2. The XRD pattern reveals peaks between 26.5°−28.16°, confirming the existence of silica content in the form of amorphous quartz. Jha, et al.^46^ also reported the maximum quartz peak at 28.6°, while studying the SCBA XRD pattern. The general diffraction angles (2θ) indicate the higher quantity of silica mainly in the form of quartz in the SCBA, demonstrating silica as the main composition, validated by XRF analysis which will assist in enhancing concrete strength. The amorphous quartz enhances the concrete strength by combining with hydration products and sustaining pozzolanic reactions under ambient temperature^47^.

In this study, the SCBA collected from Charsadda was utilized as a partial substitute material for cement in concrete production. The specific gravity and Blaine air fineness of the SCBA were 2.169 and 4720 cm^2^/gm. Furthermore, SCBA particles exhibit diverse shapes, including spherical, irregular, prismatic, and fibrous, with varying sizes^48^. The fineness and morphology of SCBA critically influence the hydration process by providing nucleation sites for CSH gel formation, densifying pore distribution, and strengthening the cementitious matrix through improved bonding at the interfacial transition zone (ITZ)^34,35^. Finer SCBA particles provide a larger surface area for more efficient hydration and CSH gel formation, leading to enhanced strength and durability. The shape of SCBA particles, particularly angular or irregular shapes, also affects how they interact with the cement matrix, contributing to the overall structural performance of the concrete^49^. Therefore, optimizing both the fineness and shape of SCBA can greatly improve the mechanical properties of SCBA-based concrete, making it a valuable material for sustainable construction practices.

#### Water

Portable water available in the concrete laboratory was used for the curing and mixing of concrete samples.

#### Aggregates

**Fig. 3:**
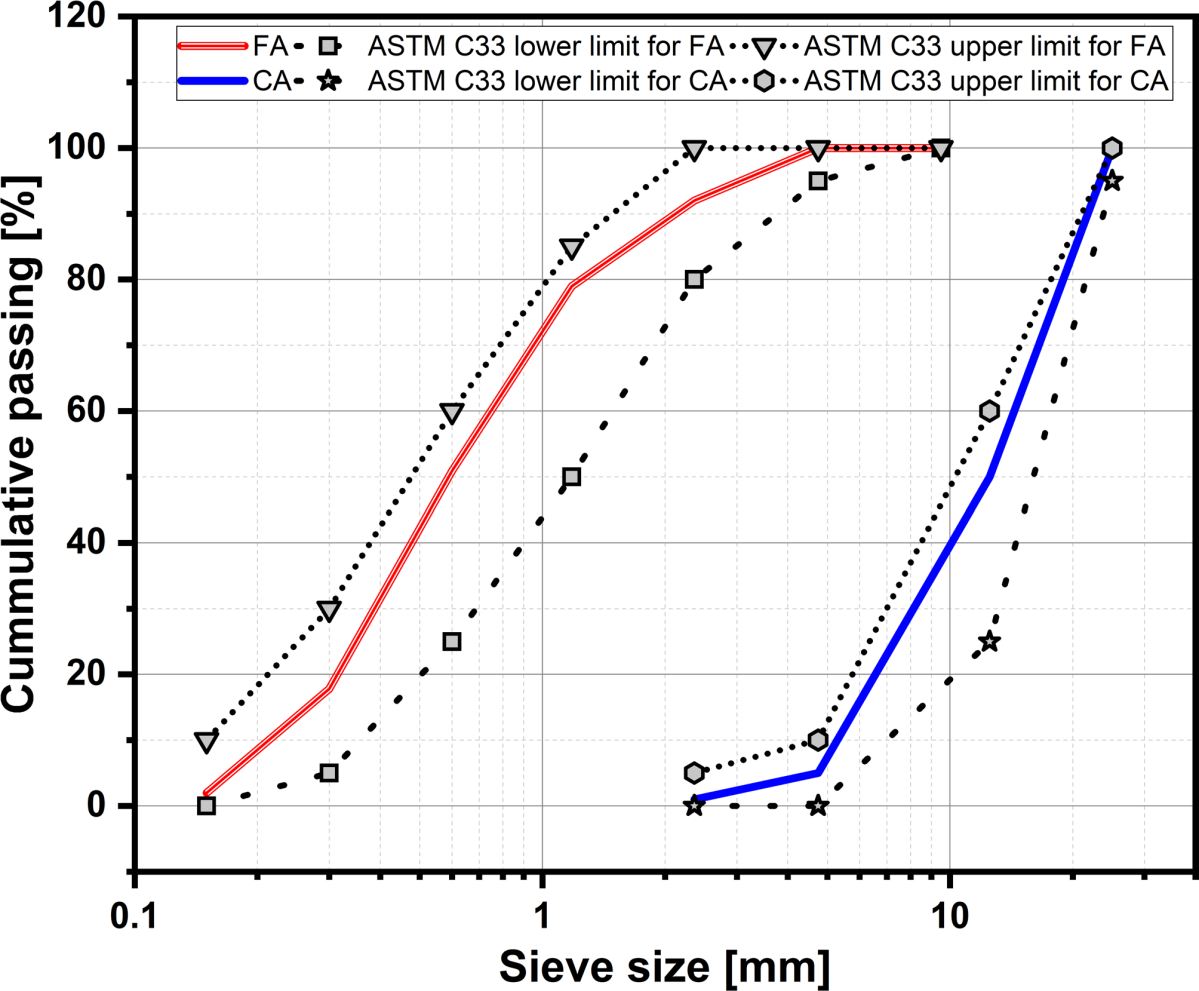
Gradation curve for fine aggregate and coarse aggregate.

Local crushed gravel and natural sand were utilized as coarse aggregate (CA) and fine aggregate (FA) to prepare concrete specimens. Figure 3 displays the gradation curves of the CA and FA, according to ASTM C136-06^50^. Following ASTM C128-22^51^, the FA had a specific gravity and water absorption of 2.61 and 1.98%, respectively, with a fineness modulus of 2.50^50^. According to ASTM C127-24^52^, the CA specific gravity and water absorption were recorded as 2.67 and 0.91, respectively.

##### Mixed proportions

In this study, overall, there are four mixes with cement replacement by SCBA of 0%, 5%, 10%, and 15%. The SCBA replacement levels (5%, 10%, 15%) were selected as per the optimal 05–15% range identified in prior studies for pozzolanic efficiency, while balancing mechanical performance and durability^31–33^. A fixed water-to-binder (W/B) ratio of 0.40 was maintained across all mixes, with superplasticizer dosage (1.0 to 1.4% by cement weight) adjusted to achieve consistent workability (target slump: 120 ± 30 mm), adhering to ASTM C192 mixing protocols^53^. The concrete mixture with no SCBA content was designated as a control mix and represented as CM. The concrete mixes containing SCBA 5%, 10%, and 15% are designated as SCBA-P05, SCBA-P10, and SCBA-P15, respectively. The total amount of OPC for each mixture was 397.8 kg/m^3^, therefore 159.12 kg/m^3^ water was kept the same for all the mixtures. The fine and coarse aggregate of 598.78 Kg/m^3^ and 1105 Kg/m^3^ were fixed for all the mixtures. Furthermore, to achieve workability with slump values of 120 ± 30 additional naphthalene-based high-range water-reducing admixture (SP) was utilized at different percentages (wt% of binder). Table 5 displays the details of the proportions of the material for each mix.

**Table 5:** Mixture proportions for control, and SCBA-based concretes.

Mix Designation	Cement (b)	SCBA	Coarse Aggregate	Fine Aggregate	W/B	SP	Slump
	Kg/m^3^					% of (b)	(mm)
CM	397.8	0	1105	598.78	159.12	1.0	120 ± 30
5% SCBA (SCBA-P05)	377.91	19.89				1.2	
10% SCBA (SCBA-P10)	358.02	39.78				1.3	
15% SCBA (SCBA-P15)	338.13	59.67				1.4	

### Testing program

#### Casting and curing

According to ASTM C192 ^**53**^ guidelines, concrete ingredients were mixed with the help of a rotatory mixer. After the required slump was achieved, the freshly mixed concrete was poured into cylindrical molds with 150 mm diameter and 300 mm height, according to the guidelines given in ASTM C-39^54^. Three identical samples for each curing day (14, 28, and 56) were prepared for compressive strength and split tensile strength of concrete, respectively. The fabricated molds were stored in a laboratory and covered with plastic sheets in a controlled environment (T = 20 ± 1 °C and RH = 60 ± 5%) for 24 h. The samples were demolded and kept in a curing tank for the specified curing ages such as 14, 28, and 56 days. Prior to testing, the top and bottom surfaces of the samples were leveled with the help of an end surface grinder.

#### Consistency and setting time

The determination of the water required to maintain standard flow and workability depends on the rheological properties of concrete. In addition, it plays a substantial role in setting hydration behavior and hardened properties of the concrete. The performance of concrete during its lifespan is primarily affected by the cement particle’s dispersion in water^55^. Considering the importance of water required for the consistency, and the initial and final setting times, the effect of various percentages of SCBA in concrete mixes was investigated using the VICAT apparatus following ASTM C-187^56^ and ASTM C-191^57^, respectively.

#### Compressive strength

Following the ASTM C-39^54^, compressive strength tests were conducted on the three identical concrete cylinder samples for each mix at the curing periods of 14, 28, and 56 days as per standards. The samples were tested under a constant load applied at a rate of 0.2 MPa/sec using a Universal Testing Machine (UTM) with a capacity of 200 tons.

#### Split tensile strength

The splitting tensile strength of the concrete samples was tested for the curing periods of 14, 28, and 56 days following the methodology described in ASTM C-496^58^. The samples were tested under a constant load applied at a rate of 1 MPa/min using a UTM having a capacity of 200 tons. The average value of the three samples for each curing age was used to calculate the splitting tensile strength.

#### Alkali-silica reactivity

Alkali-silica reactivity is considered a deleterious reaction causing major problems in concrete when the reactive silica in aggregates reacts with alkali hydroxides in the cement forming a gel that expands leading to cracks, strength loss, and durability issues. The use of SCBA in concrete effectively mitigates ASR because of its high amorphous silica content, which actively participates in pozzolanic reactions with available alkalis and calcium hydroxide in the cement matrix. Thus, producing secondary CSH-gel with a reduction in alkali content minimizes the chances of ASR-induced expansion^16^. In the current study, ASTM C 1260^59^, was adopted to evaluate the Alkali Silica Reactivity of SCBA based concrete. In accordance with the ASTM standard, tests were conducted on bars having dimensions (25 mm x 25 mm x 285 mm). The specimens were demolded after 24 h of casting and were placed in sodium hydroxide (1 M, NaOH) solution, maintaining the temperature of 80 C° ± 2 C°. The dimensional change of the specimens was recorded after 3, 7, 14, and 16 days, using an extensometer with 0.001 mm accuracy.

#### Microstructure investigation

##### Preparation of cement pastes for microstructure investigation

Control cement paste specimen and three binary pastes specimens with different SCBA percentages for cement replacement (by weight) were prepared. A Hobart mixer was employed to mix the specimens properly. The specimens were stored in circular plastic containers with dimensions of 20 mm [diameter] and 50 mm [height] after proper mixing. The containers were sealed and kept for 28 days of curing at ambient temperature. The test specimens were de-molded and dried using an ion exchange method to ensure the stoppage of the hydration process. The acquired powdered and thinly sliced specimens were cleaned in isopropanol for 15 min. After treatment, the specimens were kept in the laboratory oven at 40 ℃ for 30 min. The specimens obtained were held in polythene plastic bags till testing.

##### X-ray diffraction (XRD)

The mineralogical investigation of well-ground cementitious specimens was placed into a specially designed sample handler for analysis using X-ray powder diffraction (XRD, JDX 3532, JEOL, Japan, with SX-Rays-Cu Ka (I = 1.5418 Å). The XRD data were obtained in the range of 2θ = 0° to 80°, installed in Central Resource Labs (CRL), University of Peshawar. After 28 days, the cured samples were taken out from their molds and were crushed in smaller fractions which were treated with ethanol to stop further hydration^60^. Before analysis, the smaller fraction was further oven-dried and grounded to a particle size lower than 70 μm^61^.

##### SEM-EDX

The microstructural behavior of cement pastes was studied using the SEM-EDX technique on thick slices prepared from the hardened specimens with JSM-IT100 installed in Central Resource Labs (CRL), University of Peshawar. As discussed above, the solvent exchange procedure was adopted for drying the hardened paste slices with the help of isopropanol. The microstructural and compositional transformations of the tested specimens were discussed.

##### Nitrogen adsorption isotherm technique

Besides XRD and SEM analysis, N_2_ adsorption tests were conducted on the powdered samples (weighing approximately 0.3 g) obtained from the control mix and SCBA-based mixes after 28 days of casting. An N_2_ adsorption analysis was performed to find the induced pore volume and BET surface area using (NOVA2200e, Quanta chrome, USA) analyzer, maintaining the temperature of 273 K, at Central Research Laboratory, University of Peshawar. During the procedure, first the powdered samples were degassed to remove contaminants absorbed from the air. Similarly, the test was performed maintaining controlled pressure and ambient temperature.

##### Environmental assessment and economic feasibility

**Fig. 4:**
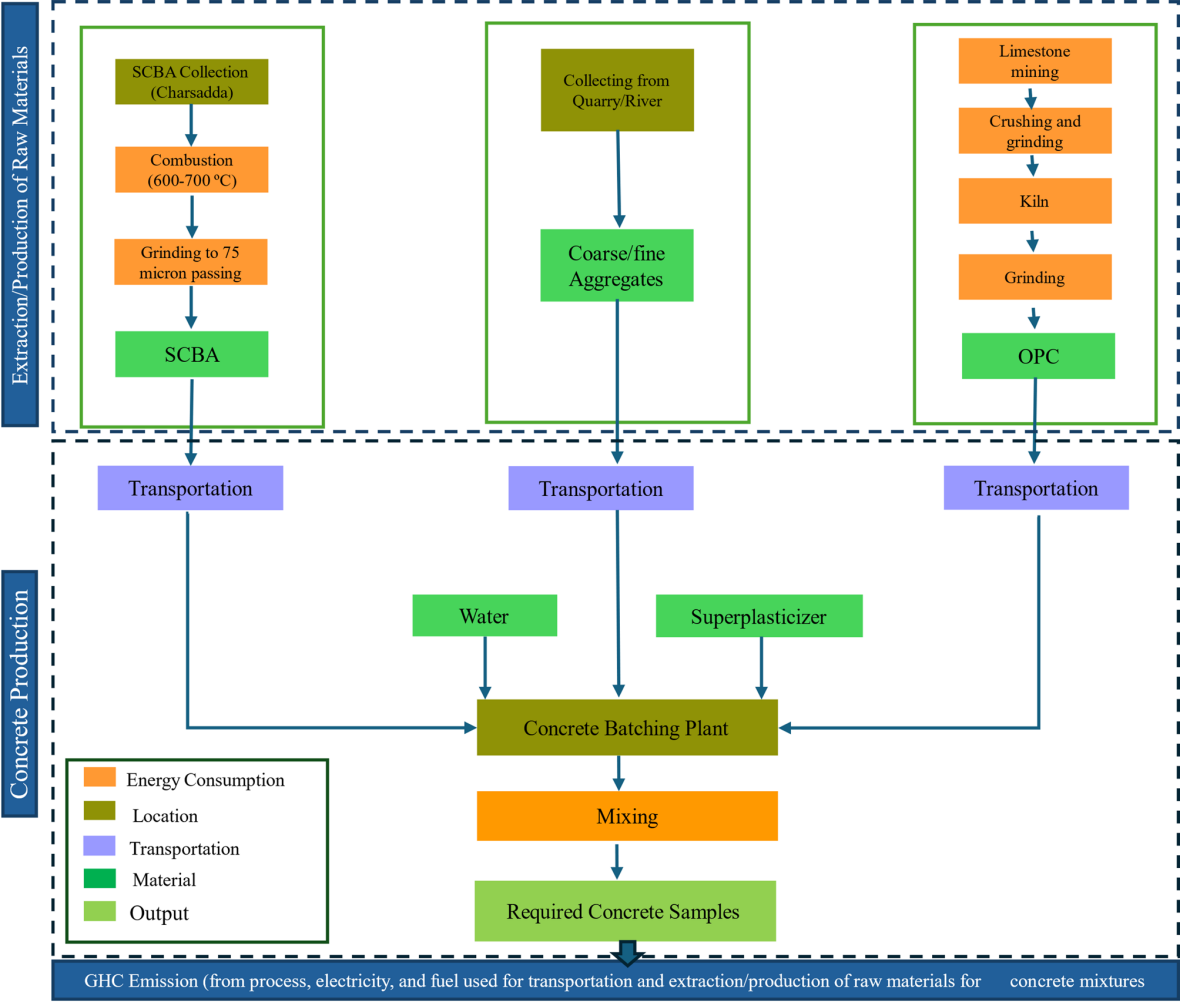
The system boundary for carbon emission for control and SCBA-based mixes.

The carbon emissions and cost analysis for SCBA-based concrete was performed to examine the environmental effects and financial feasibility of the concrete compositions, which will help civil engineers and designers in the application of SCBA-based concrete in construction projects. The current study examines the cradle-to-gate phase, which includes raw material extraction, processing, and concrete production. The assessment excludes transportation to construction sites, the construction phase itself, and end-of-life disposal, aligning with the boundaries established in prior research^62^. The system boundaries for manufacturing both CM and SCBA-based concrete are illustrated in Fig. 4. The estimation procedures are discussed in detail in Sect. 3.9.

## Presentation and discussion of results

### Consistency

Table 6 demonstrates that the quantity of water required for standard consistency linearly increases with a percentage increase in substitution of SCBA compared to the CM. The primary reasons for the rise in water demand in the SCBA-based paste are most probably due to the low specific gravity and porous nature of the SCBA particles and higher surface area^6,63^. Furthermore, the rough surface and high porosity of SCBA increase the water adsorption into the particles, and therefore high amount of water will be required to lubricate the particle’s surfaces^64^. In the current study, the SCBA has a specific gravity of 2.16 with a specific surface area of 4720 cm^2^/g. Therefore, it provides evidence that SCBA has fine particles and low specific gravity compared to OPC and needs more water to achieve the desired consistency. The values of normal consistency increased by 10%, 14.28%, and 21.66% for the SCBA-P05, SCBA-P10, and SCBA-P15 samples compared to the control cement paste. The interaction between the SCBA’s specific area and having a porous nature influences the water demand significantly. High surface area leads to higher demand for water, but if SCBA has significant porosity, it will absorb more water, resulting in increased water requirement to achieve a workable mix^49^.

**Table 6:** Consistency and initial setting times (IST) and final setting times (FST) of control mix and SCBA-based pastes.

Mix Designation	Consistency (%)	IST (min)	FST (min)
CM	30	105	256
SCBA-P05	33	121	268
SCBA-P10	35	129	281
SCBA-P15	36.5	142	297
AS3972 limits		≥ 45	≤ 600

*P: percentage replacement.

### Setting time

Table 6 demonstrates the values of the initial and final setting times of CM and concrete mixtures containing SBCA. The IST and FST increase with the increase in the amount of SCBA. The increase in IST and FST values may be because of the decrease in cement proportion. The FST values increased by 9.76%, and 16.01% for the SCBA-P10% and SCBA-P15 as compared to the CM. The rise in IST and FST values utilizing SCBA was previously noticed^6^. In addition, all the setting times values were within the range given by AS-3972^65^ which states an IST value of greater than or equal to 45 min and an FST value lower than 600 min. Lohtia and Joshi^66^ reported that adding silica fume in concrete with no water-reducing admixture delays the setting time compared to conventional concrete. The lack of a water-reducing agent may be responsible for the increase in setting times for SCBA cement pastes compared to conventional paste. Notably, research has shown that incorporating SCBA may unexpectedly extend the concrete’s setting time. This phenomenon, consistent with findings by Wang, et al.^67^ occurs due to delayed hydration reactions caused by SCBA addition. Balancing these characteristics is crucial for optimizing the performance of SCBA-based concrete, as it impacts both the initial workability and long-term durability.

### Effect of SCBA on the compressive strength of concrete against curing ages

**Fig. 5:**
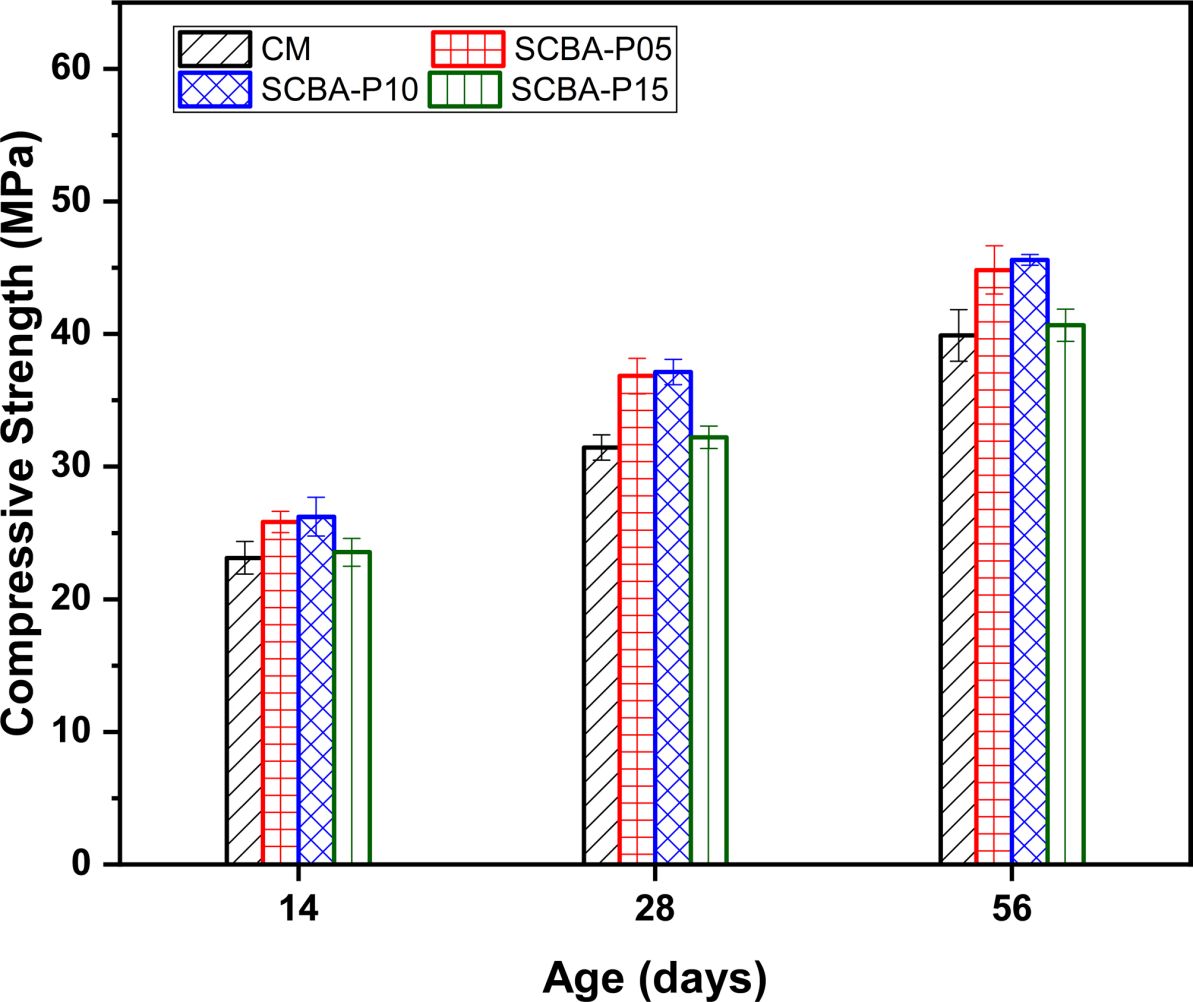
Compressive strength of cement mix and SCBA-based mixes with different percentages.

Figure 5 displays the results of compressive strength obtained for the Control Mix and SCBA-based samples (SCBA-P05, SCBA-P10, and SCBA-P15) after 14, 28, and 56 days of curing. To ensure data reliability and validate the observed trends, each experimental result represents the mean value of three measurements. Standard deviations (SD) were calculated for all compressive strength tests at 14, 28, and 56 days. The experimental test results revealed that the concrete mixes, at all percentages SCBA replacements 5%, 10%, and 15%, displayed a noticeably better compressive strength than the CM at all curing ages. It can be observed that the CS development is higher for the SCBA replacement up to 10% and reduced with an increase in the SCBA of 15%. The increase in CS with the incorporation of SCBA up to 10% could pertain to a high percentage of silica reacting with calcium hydroxide and water resulting in CSH gel^68^. The reduction for the 15% SCBA may be due to the decrease in the amount of OPC and the higher porosity nature of the SCBA^69^. Furthermore, the low reactivity of SiO_2_, decrease in CaO contents and slow hydration reaction also tend to reduce the CS^32^. However, the findings revealed that CS increases for the SCBA-P-05, SCBA-P10, and SCBA-P15 SCBA substitution compared with CM at 28 days, respectively. The improvement in compressive strength is attributed to the high pozzolanic reaction and high amount of amorphous silica. Although, the substitution of up to 15% of SCBA still enhances the high strength of concrete in comparison to CM. The addition of SCBA certainly produces comparatively high-strength concrete because of its finer particles, high silica content, and improved surface area resulting in number of nucleation sites for further hydration products^31^. The increase in the CS for SCBA-P05, SCBA-P10, and SCBA-P15% mixes compared to CM was 17.74%, 18.71%, and 3.87%, at 28 days, respectively. For instance, on 14 and 28 days, the SCBA-P05 sample strength was higher compared to CM by 11.69% and 17.74% respectively for the age of 14 and 28 days. Similarly, the CS of the SCBA-P10 concrete was greater than the CM by 13.42% and 18.71%, respectively. The same trend was followed by SCBA-P15 with an increase of 1.73% and 3.87% on 14 and 28 days, respectively, slightly larger than the CM. Additionally, the increase in CS development from 14 days to 28 days was remarkably higher for SCBA-P10 compared to SCBA-P05. The results also showed that at 56 days of age, the strength increase for the SCBA-P05, SCBA-P10, and SCBA-P15 samples were 12.28%, 14.29%, and 1.75%, respectively, higher than CM. The low early CS and enhanced later-age CS are common characteristics of pozzolans, such as SCBA^32,69^. The CS results indicated that 10% of SCBA is an optimal limit for replacement of OPC.

### Effect of SCBA on the split tensile strength of concrete with curing ages

**Fig. 6:**
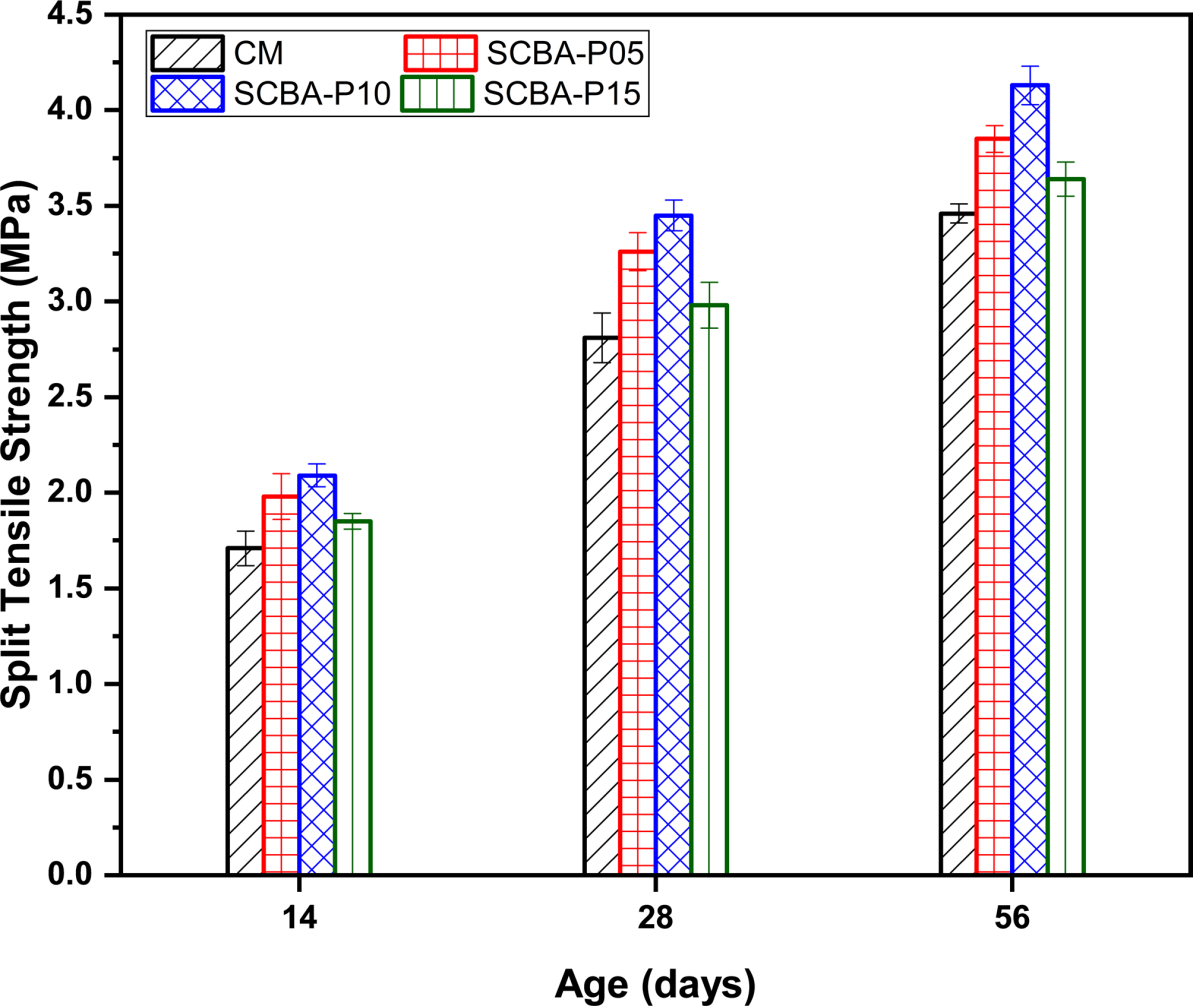
Split tensile strength of control mix and SCBA-based mixes with different percentages.

Figure 6 presents the results of split tensile strength for CM and SCBA-based concrete. To ensure data reliability and validate the observed trends, each experimental result is presented as the mean value of three measurements. Standard deviations (SD) were calculated for all split tensile strength tests conducted at 14, 28, and 56 days. The specimens containing SCBA showed a considerable increase in split tensile strength at all test ages in comparison to CM. Similar to compressive strength, both SCBA-P05 and SCBA-P10 mixtures exhibited significantly higher split tensile strength compared to SCBA-P15 and CM, irrespective of aging.

**Fig. 7:**
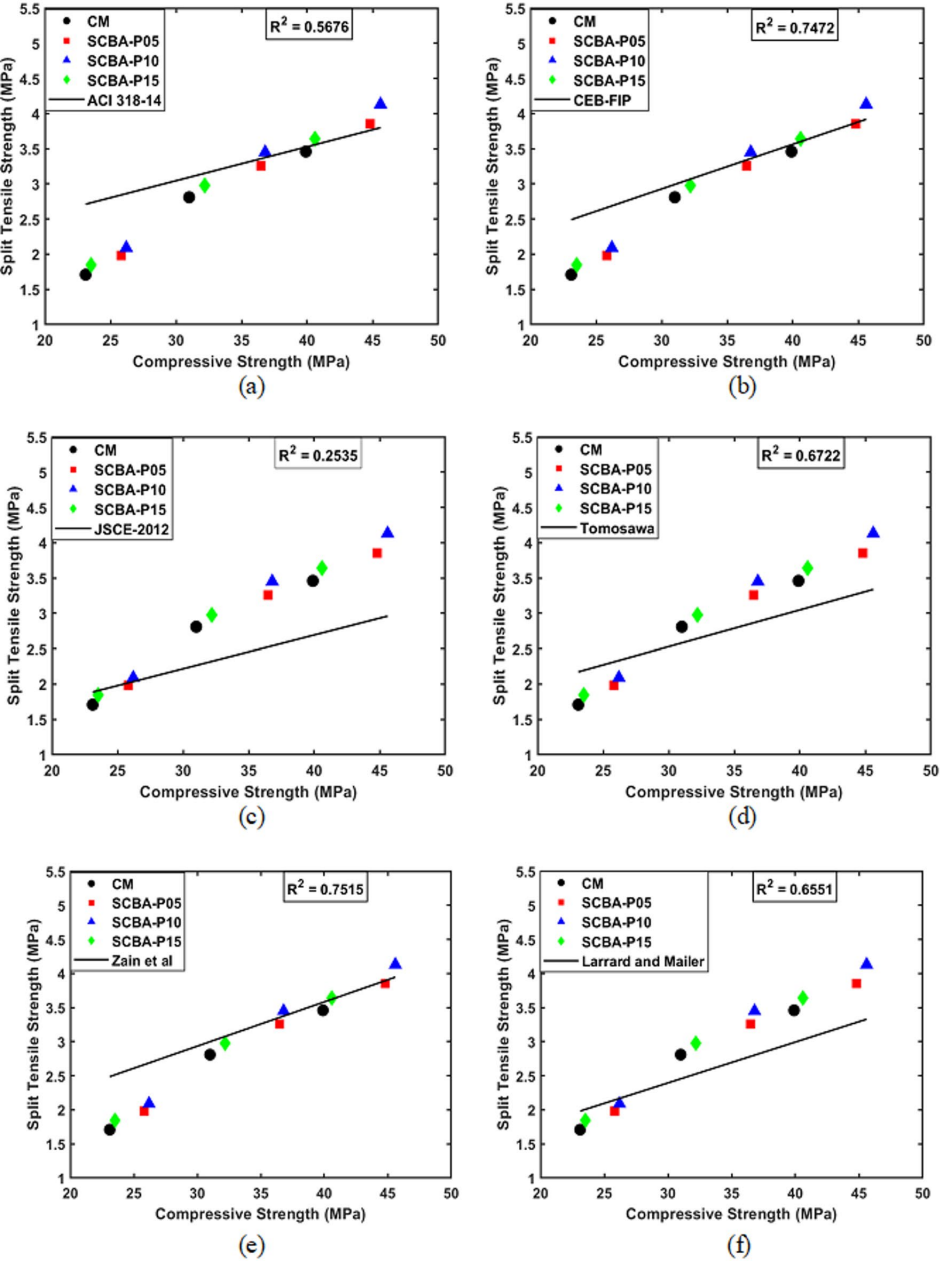
Correlation between compressive and tensile strength of concrete: experimental results with different proposed model equations.

The splitting tensile strength values for the SCBA-P05, SCBA-P10, and SCBA-P15 after the age of 14 days were 13.79%, 20.11%, and 6.32% higher than CM, respectively. The significant increase in CS of SCBA-based mixtures at early ages suggests that SCBA enhances early hydration reactions. SCBA having a pozzolanic nature reacts with calcium hydroxide (formed during cement hydration) resulting in additional CSH gel, which contributes to increased strength, a denser and more refined microstructure, and improved interlocking between cement and aggregates^68^. A similar trend was observed in the splitting tensile strength values at 28 days of age, with SCBA-P05, SCBA-P10, and SCBA-P15 showing increases of 16.01%, 22.78%, and 6.05%, respectively, compared to CM. Likewise, at 56 days of age, the percentage increases in splitting tensile strengths were 11.27%, 19.36%, and 5.20%, respectively. The improved tensile strength at 28 and 56 days of curing age might be attributed to the long-term strength development driven by SCBA’s pozzolanic activity, which continues to produce additional CSH gel^69^. The decrease in splitting tensile strength for SCBA-P15 compared to SCBA-P05 and SCBA-P10 could potentially be attributed to the existence of a substantial proportion of unreacted SCBA particles. These particles may not fully participate in the pozzolanic reaction, thereby limiting their contribution to strength development beyond the optimal percentage^70^. Various standards and researchers have proposed empirical equations to describe this relationship, which are summarized in Table 7. Figure 7 presents a comparison of experimental data with predictive models from various codes and researchers. The results revealed that the proposed equation by Zain et al.^71^ provides the most accurate prediction achieving a coefficient of determination (R^2^) of 0.75. this is closely followed by CEB-FIP^72^which achieves an R^2^ value of 0.74.

**Table 7:** Comparison between compressive and splitting tensile strength of concrete with existing prediction equations.

Model code	Model equation	Values of	
		a	b
ACI 318–2014^73^	$f_{sp}=a\:(f^\prime\:c)^b$	0.56	0.50
CEB-FIP^72^		0.301	0.67
JSCE-2012^74^		0.23	0.667
Tomosawa et al.^75^		0.291	0.637
Zain et al.^71^	$f_{sp}=f^\prime\:c/[a(f^\prime\:c)+b]$	0.10	7.11
Larrard and mailer^76^	$f_{sp}=a+b(f^\prime\:c)$	0.60	0.06

**Fig. 8:**
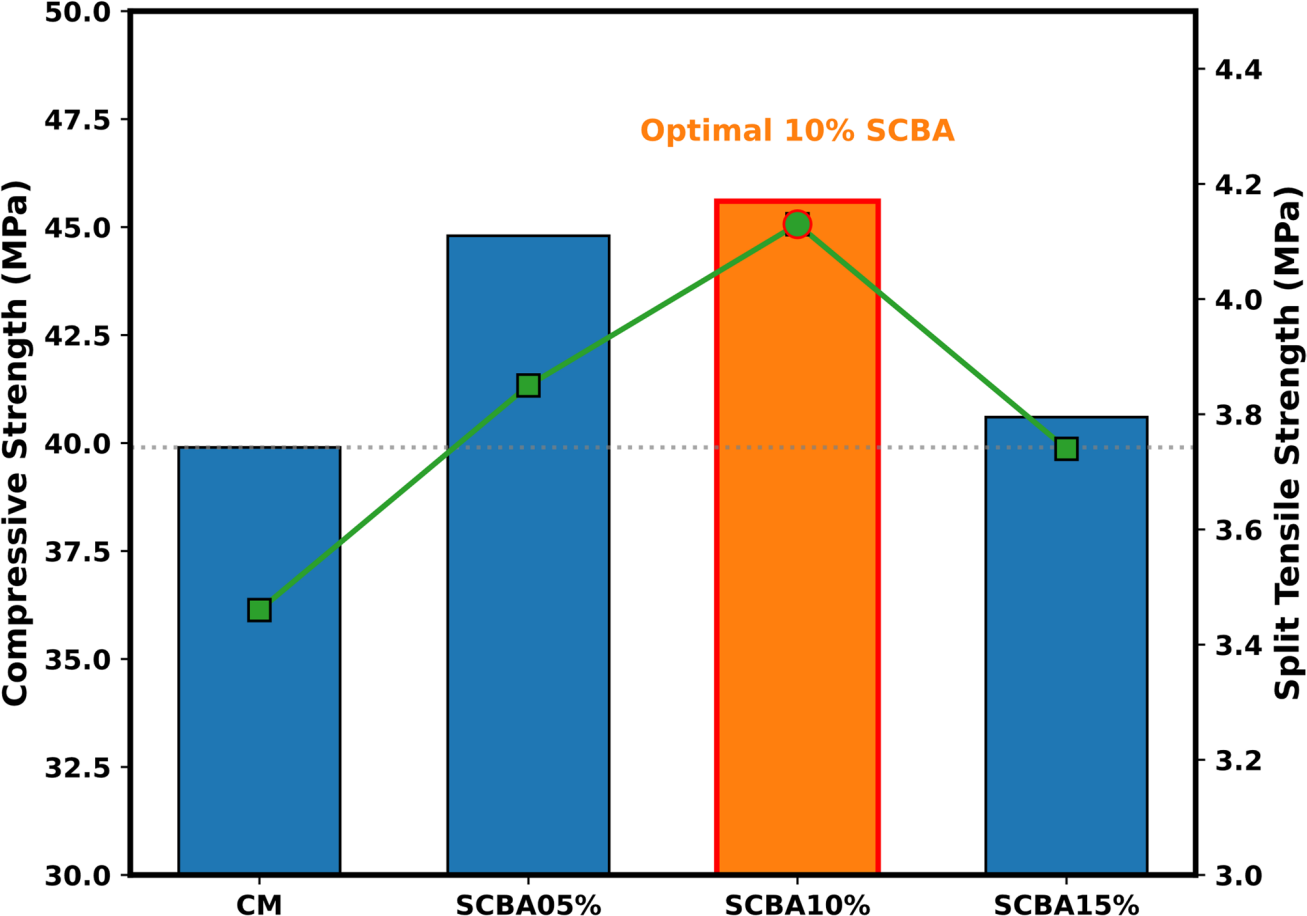
Effect of SCBA replacement on compressive and split tensile strength: identification of optimum level.

In summary, a remarkable improvement in both CS and Split tensile strength was observed by the 10% SCBA mix compared to all other concrete mixes, as displayed in Fig. 8. The results align with previously reported findings, which also demonstrated enhancement in both CS and split tensile strength for the 10% SCBA mix compared to other specimens^77^. Sobuz, et al.^78^ also observed the same findings with lower substitution of SCBA the split tensile strength increased, this increase is attributed to the granularity of SCBA resulting in rapid reaction between silica and Ca(OH)_2_.

### Effect of SCBA on the expansion due to alkali-silica reactivity

**Fig. 9:**
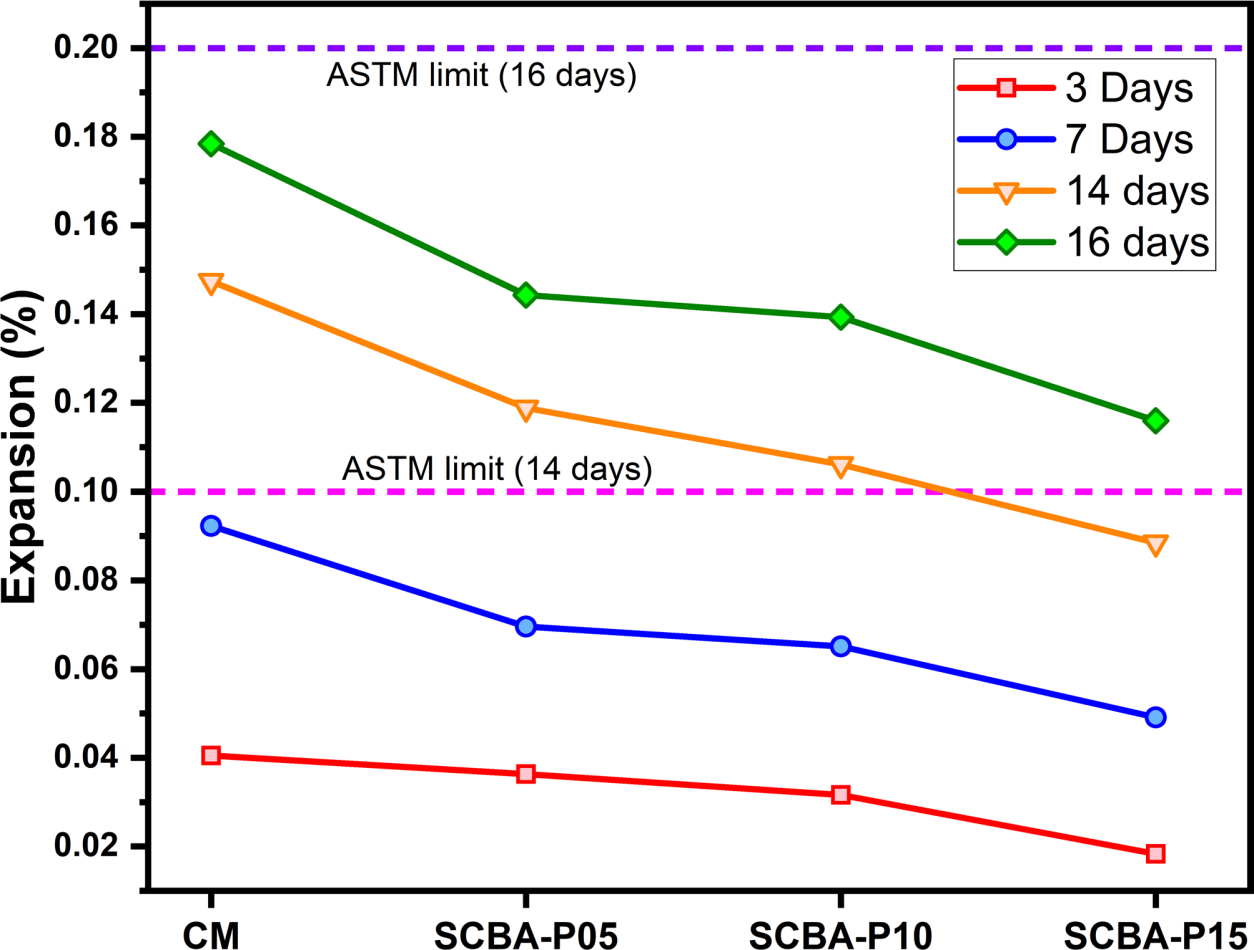
ASR results for CM and SCBA-based concrete with different percentages.

Figure 9 displays the expansion results of the concrete bar specimens used to evaluate the Alkali silica reactivity of SCBA based concrete. Following ASTM C-1260, all specimens CM, SCBA-P05, SCBA-P10, and SCBA-P15 represent good behavior due to the expansion which is less than 0.2% for all replacement levels after 16 days of casting. Despite the high content of silica available in the SCBA, the incorporation of SCBA mitigates the ASR with the reduction in expansion of the bars for all the replacement levels^30^. A similar trend was noticed for ASR results of RHA by Zerbino et al.^79^.

Although some pozzolanic materials such as SCBA have a high amount of silica compared to cement but still encourage a decrease in the number of alkalis in the solution. This reduction is due to the portlandite consumption during the pozzolanic reactions, decreasing the number of hydroxyls available for the ASR resulting in the formation of more CSH and mitigates expansion^80^. SCBA’s high amorphous silica content reacts with calcium hydroxide (CH) from cement hydration, producing additional CSH gel. This reaction reduces alkali availability, minimizing alkali-silica reaction (ASR) with reactive aggregates like quartz. The formation of additional CSH densifies concrete, lowering porosity and improving durability by resisting water ingress^16,80,81^. Thus, SCBA mitigates ASR by consuming CH, reducing alkalis, strengthening the matrix, and improving long-term performance in harsh conditions.

### XRD analysis

**Fig. 10:**
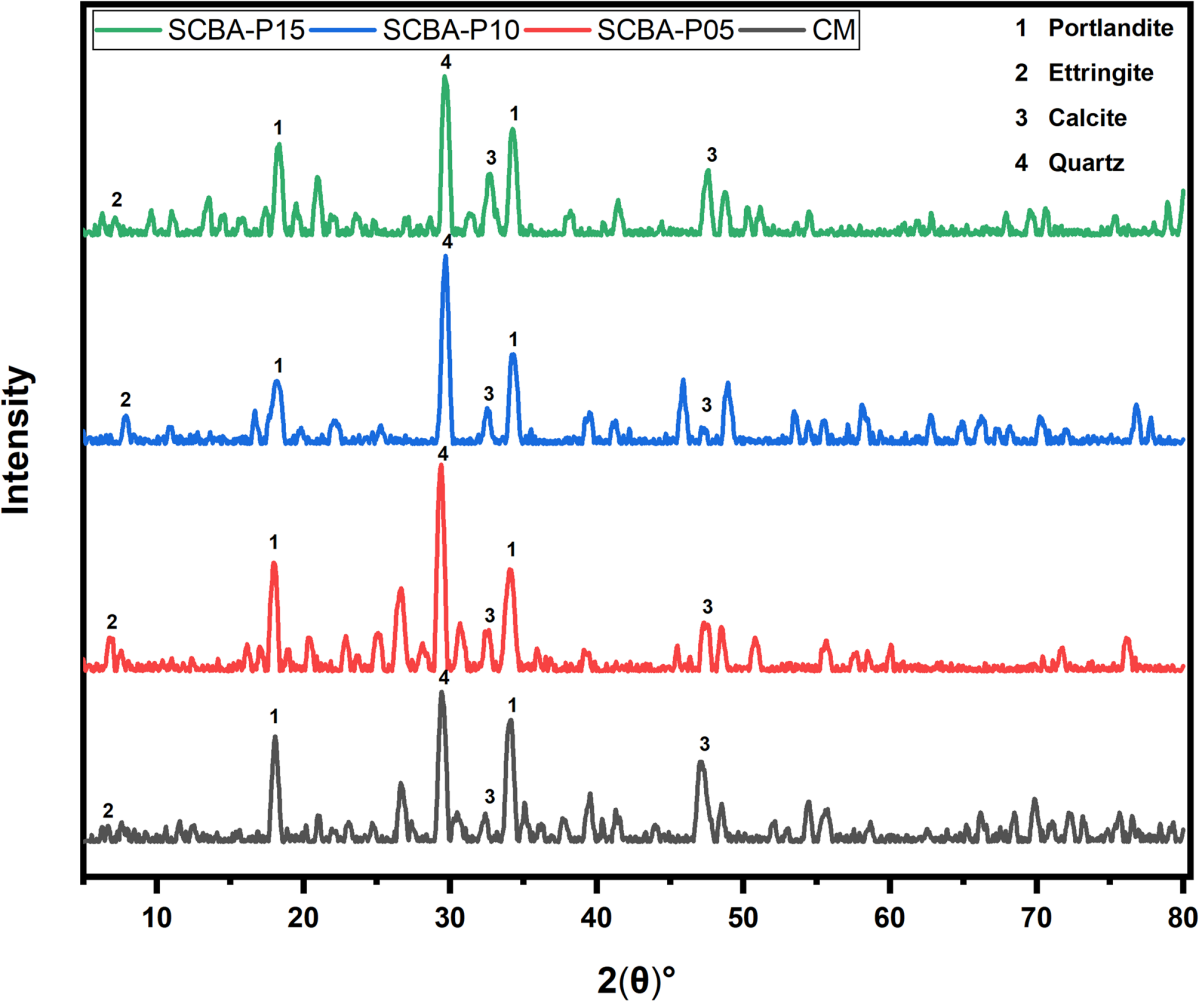
XRD patterns for control control mix and SCBA-based concrete with different percentages after 28 days.

The XRD analysis of the CM and specimens containing SCBA is shown in Fig. 10. The CM sample is represented with 0% replacement. The XRD data contain mainly portlandite, ettringite, silicates, and quartz. It has been noted that the intensity of the portlandite (Ca(OH)_2_) peaks has been reduced for a higher proportion of SCBA^70^. However, the decrease in the portlandite in all SCBA-based samples is due to the formation of a higher amount of additional CSH gel, formed by the chemical reaction between silica and Ca(OH)_2_. The reduction in portlandite peaks and increased CSH gel formation are consistent with the expected pozzolanic behavior of SCBA, resulting in improved early and long-term strength and durability of concrete. This reduction in portlandite peak is more dominant in the SCBA-P10, illustrated by its higher mechanical properties. These results show the production of high-density CSH-matrix by adding 10% of SCBA as a cement replacement. Portlandite consumption increases with the incorporation of SCBA, which indicates SCBA’s high pozzolanic reactivity as mentioned in Fig. 10. The peak of quartz confirms the occurrence of crystalline silica. The presence of calcite peak suggests the reaction between CH and pozzolanic materials in the presence of water has occurred, resulting in reduced calcium hydroxide compositions and contributing to improved concrete strength and durability^55^. Moreover, the inclusion of SCBA in the SCBA-P10 mix leads to a reduction in calcium hydroxide (Ca(OH)₂), a phase known for its relative weakness and porosity. This decrease in Ca(OH)₂ peak contributes to a more compact microstructure, as SCBA facilitates the formation of additional CSH phases, enhancing the overall performance of the concrete^82^. The clear, well-defined peaks observed in the XRD analysis indicate the presence of crystalline structures with organized atomic arrangements. This characteristic is essential for ensuring the mechanical strength and stability of the concrete^83^.The results obtained from the XRD graph align with SEM results providing evidence of high consumption of portlandite resulting in the formation of more high-density CSH gel for all percentages of SCBA substitutes.

### SEM-EDS analysis

**Fig. 11:**
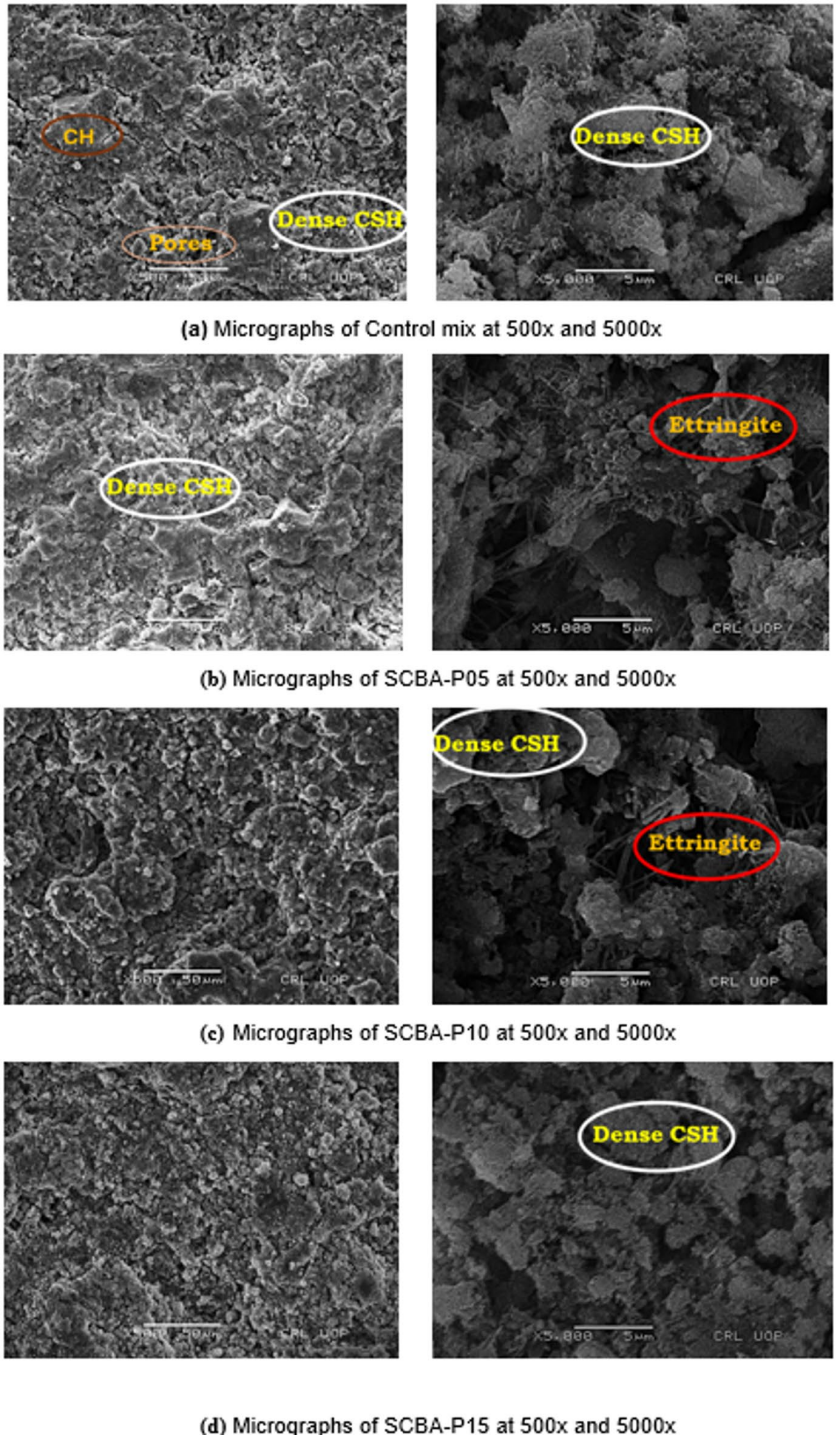
SEM micrographs control mix and SCBA-based concrete with different percentages after 28 days: (**a**) Control Mix; (**b**) SCBA-P05; (**c**) SCBA-P10; (**d**) SCBA-P15.

The results of SEM micrographs for the CM and SCBA-based concrete samples are demonstrated in Fig. 11, respectively. These micrographs present the impact of SCBA as a substitution of cement with different percentages. The purpose was to determine the microstructural composition of the cement paste matrices, which are considered a crucial parameter for analyzing the performance of mineral admixtures utilized in cement for designing high-strength and sustainable concrete with reduced carbon emissions. The chemical composition of hydration products like CSH and CH mostly depends upon the chemical reactivity of Calcium and Silicon ions present in the cement pore solution at the time of hydration reaction^84^.

The microscopic study illustrates that SCBA-P05, SCBA-P10, and SCBA-P15 samples exhibited a denser and more compacted matrix compared to the control mix, as shown in Fig. 11. These results may be related to SCBA’s high reactivity, which produces high-density hydrates, and ettringite formation^85^. The pozzolanic reaction of SCBA in the cement matrix mainly involves the consumption of calcium hydroxide (Ca(OH)₂), a product of cement hydration, which leads to the formation of additional calcium silicate hydrate (CSH) gel. During cement hydration, the C₃S and C₂S phases release Ca(OH)₂ and CSH. The reactive amorphous silica in SCBA reacts with the released Ca(OH)₂, as shown in the simplified reaction elaborated as Eq. 1^86^.

1 $$\text{Si}\text{O}_{2}+\text{Ca}(\text{OH})_2+\text{H}_2\text{O}\rightarrow\:\text{CSH}$$

The samples with SCBA up to 10% replacement displayed denser and well-defined microstructure, resulting in improved compressive strength after 28 days of age. This improved microstructure might be attributed to the reaction of silica from SCBA with CH forming additional CSH gel^87^. Furthermore, the microstructure of SCBA-based samples revealed needle-like structures as highlighted, which might be attributed to the formation of ettringite^88^. The higher percentage replacement of SCBA forms a dilution effect of cement due to increased silica content resulting in a high number of unreacted particles of SCBA. Thus resulting in CS reduction compared to low percentages of SCBA substitution^89^. The micrograph results agree with the XRD observations showing the high ettringite peaks compared to the CM.

**Fig. 12:**
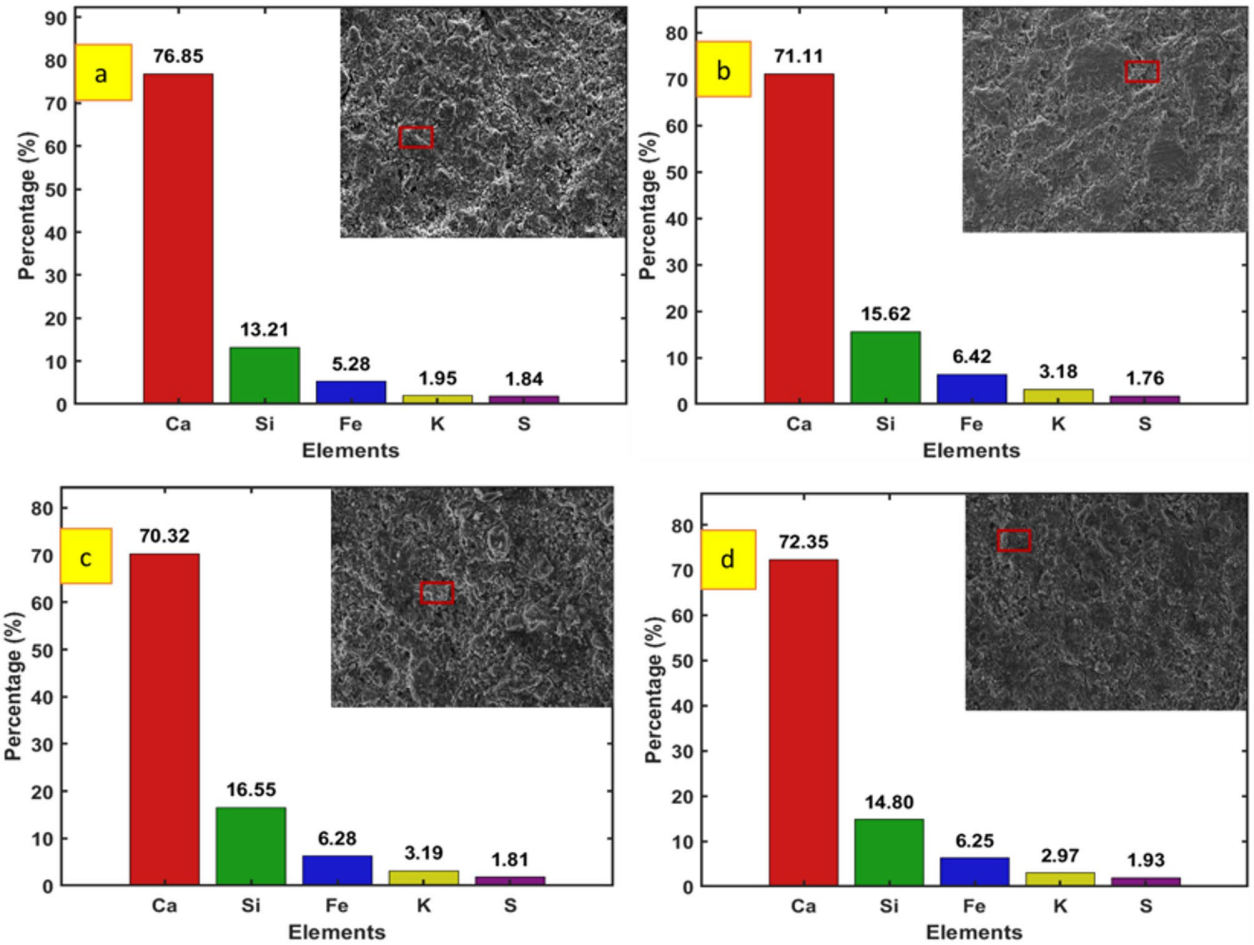
EDX graphs for control paste and SCBA-based pastes with different percentages after 28 days: (**a**) Control Mix; (**b**) SCBA-P05; (**c**) SCBA-P10; (**d**) SCBA-P15.

The elemental compositions of CM and SCBA-based samples are shown in Fig. 12, respectively. From the results, the value of Ca for the sample SCBA-P10 was lower compared to all the respective samples. The reduction in Ca content is mainly due to the consumption of more Ca for forming CSH gel. The Ca/Si value for the CM is high compared to all other samples. With the increase in SCBA percentages, the Ca/Si value decreases which indicates that additional silica from SCBA reacts with CH to produce more CSH gel^90^. The results are in line with the observations concluded from the compressive and split tensile strength results where the SCBA-P10 samples show higher strengths compared to other concrete mixes.

### N_2_ adsorption

Table 8 compares the results obtained for the induced pore volume for all the samples (CM, SCBA-P05%, SCBA-P10%, and SCBA-P15%) after standard curing of 28 days. According to the results obtained SCBA-P10% (0.052 cm^3^/g) exhibited the lowest induced pore volume compared to all test specimens. This is attributed to SCBA’s improved high pozzolanic reactivity, resulting in densification of pore structure^91^.

**Table 8:** Induced pore volume results of control mix and SCBA-based pastes.

Mix designation	Induced pore volume (cc/g)
CM	0.056
SCBA-P05	0.055
SCBA-P10	0.052
SCBA-P15	0.055

*P: percentage replacement.

Table 9 demonstrates the BET surface areas of the control sample and SCBA-based samples. According to the results, SCBA-P10% exhibited the highest surface area followed by SCBA-P5%, SCBA-P15%, and CM. Previous researchers reported that an increase in BET surface area suggests the improved microstructure of CSH gel^92^. This was also demonstrated by XRD and SEM analysis of the formation of a dense matrix with the formation of more CSH gel.

**Table 9:** Results of BET surface area between control and SCBA-based pastes.

Mix designation	BET surface area (m^2^/g)
CM	11.7
SCBA-P05	12.79
SCBA-P10	13.08
SCBA-P15	12.39

*P: percentage replacement.

### CO_2_ emissions and cost analysis

The findings achieved in this study verified that the utilization of SCBA enhanced the mechanical properties and microstructure of concrete. Besides improving those properties, concrete comprising the SCBA was assessed in terms of eco-friendly material regarding CO_2_ emissions and financial viability. The cost and CO_2_ emissions of concrete materials are listed in Table 10. The production of cement emits 820 kg-CO_2_/ton, while SCBA is regarded as a waste product and therefore releases less CO_2_ than cement. Transportation is considered to be the primary cost of SCBA. The expenses of grinding involve equipment and labor costs, and energy consumptions and are 30% of the overall cost of the SCBA^3^. The local use of the SCBA further decreases the costs of SCBA. The cost and CO_2_ emissions of SCBA is 4.5 and 7.45 times less than OPC. The cost of SCBA is much lower than cement because it is not utilized and mostly remains as waste for discarding with no advantage. The utilization of SCBA decreases the costs and also decreases the accumulation load on landfills.

**Table 10:** Cost and carbon emissions of concrete materials.

Materials	CO_2_ emission (kg-CO_2_/ton)	Unit cost (USD)
Portland cement	820^a, b^	64.7 ^a, b^
SCBA	110 ^a, b^	15 ^a, b^
Fine aggregate	28 ^a, b^	13.2 ^a, b^
Coarse aggregate	39 ^a, b^	16.2 ^a, b^
water	0.8^c^	2.1^c^

a Data taken from Chindaprasirt, et al.^3^.

b Data taken from Li, et al^27^..

c Data taken from Nakararoj, et al^93^.

**Fig. 13:**
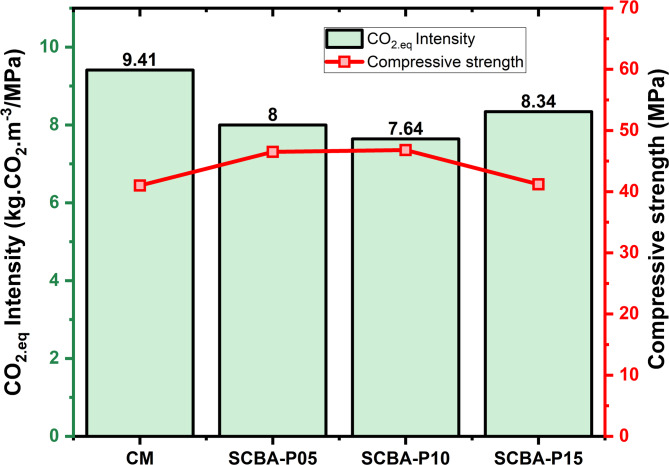
Comparison between CO_2_-eq emission and CS values at 28 days.

Figure 13 presents the comparison of the CO_2_ emissions and CS values at 28 days. Usually, CS values at 28 days are considered for comparison purposes. The CO_2_ emissions values for the concrete mixes CM, SCBA-P05, SCBA-P10, and SCBA-P15 were 9.41, 8.00, 7.64, and 8.34 kg.CO_2_.M^−3^/MPa, respectively. The findings revealed that CO_2_.eq values of concrete comprising a large amount of SCBA were less as compared to the CM. The results showed good agreement with the previously achieved findings^3^. The SCBA-P15 concrete resulted in the lowest CO_2_ emissions. However, the reduction in CS value was noted for SCBA-P15. The SCBA-P10 concrete significantly reduced the CO_2_ emissions and enhanced the CS values in comparison to CM and could develop a sustainable eco-friendly concrete.

**Fig. 14:**
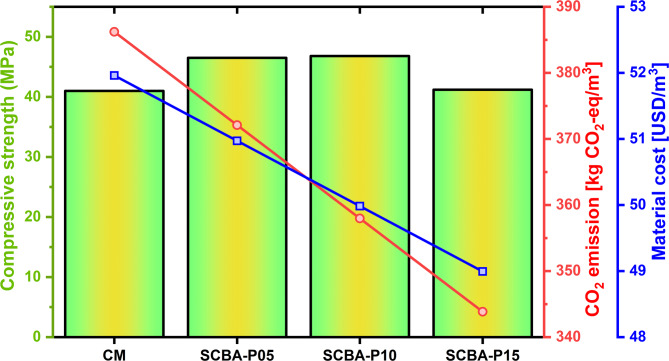
Comparison between CO_2_ emissions and cost reduction and CS values at 28 days.

Figure 14 presents the comparison of cost and CO_2_ emissions values against corresponding CS values at 28 days. The results demonstrate that cost and CO_2_ emissions are reduced by increasing the SCBA’s percentage in the concrete mixes. The CO_2_ emissions were reduced by almost 3.80%, 7.89%, and 12.32% for the SCBA-P05, SCBA-P10, and SCBA-P15 respectively as compared to the CM. The cost of concrete mixes SCBA-P05, SCBA-P10, and SCBA-P15 decreased by approximately 1.94%, 3.96%, and 6.05% respectively in comparison to the CM. The addition of suitable content of SCBA enhanced the strength of concrete as well as cost-saving and environmentally friendly. The findings suggest that incorporating 10% SCBA (SCBA-P10) as a partial cement replacement in concrete is the most favorable option in terms of strength, environmental benefits, and cost-effectiveness. In summary, the use of SCBA in concrete production offers significant financial advantages through cost reductions in raw materials and disposal costs, while also contributing to substantial CO₂ emissions reductions^49^. SCBA’s potential for industrial-scale utilization is significant due to its abundant availability as a by-product of sugarcane processing, particularly in sugar-producing regions. Scalable production of SCBA could easily meet the growing demand for sustainable construction materials. Additionally, the industrial-scale utilization of SCBA is not only feasible but also offers the opportunity to further enhance both the economic and environmental benefits of concrete production^94^. This makes SCBA a cost-effective and sustainable alternative to conventional cement, aligning with the global drive toward sustainable construction practices and environmental responsibility.

## Conclusions

In the current study, the locally available SCBA with different percentages was utilized as a partial substitute for cement. The performance of SCBA blended cement concrete was evaluated through consistency, setting time, CS, split tensile strength, ASR, microstructure, mineralogy, elemental analysis, environmental effect, and cost feasibility. The main conclusions drawn from the experimental findings are outlined below:

- The chemical compositions of SCBA vary depending on the source due to geological, topographical, and climate factors. Based on the categorization and comparison of the oxide compositions of SCBA collected from the different areas, i.e., Malakand, Charsadda, and Mardan districts of KPK, the sample gathered from Charsadda shows the highest percentage of SiO_2_ (62.839%).
- The normal consistency is enhanced as the percentage of SBCA substitution increases in concrete. The high surface area and low specific gravity of the SCBA led to higher water demand for standard flow in comparison to OPC. The concrete mix of higher substitutions of SCBA such as 10% and 15% led to an increase in FST of 9.76% and 16.01%, respectively, compared to the OPC paste.
- The incorporation of SCBA in concrete significantly increased the CS and split tensile strength as compared to the CM at all curing ages. The 28 days CS of the concrete mix CM, SCBA-P05, SCBA-P10, and SCBA-P15 were 31, 36.5, 36.8 and 32.2 MPa, respectively. The 28-day split tensile strength of SCBA-P05, SCBA-P10, and SCBA-P15 increased by 16.01%, 22.78%, and 6.05% respectively as compared to the CM. The CS and split tensile strength results suggest that at 10% OPC substitution, the SCBA is potentially highly pozzolanic as compared to other replacement levels.
- The ASR results indicated a reduction in expansion for all SCBA-based samples compared to the CM. This reduction is due to the portlandite (Ca(OH)_2_) consumption in the pozzolanic reactions.
- XRD analysis results indicated that the intensity of the Ca(OH)_2_ has been reduced with the increase in the percentage of SBCA contents. The reduction in the Ca(OH)_2_ in the SCBA-based concrete may be due to the formation of additional CSH gel, formed by the reaction of reactive silica with Ca(OH)_2_. The formation of CSH gel is more dominant in the 10% replacement, also confirmed by their higher mechanical strength.
- The EDX characterization and SEM micrographs showed a decrease in Ca values for all SCBA-based concrete in comparison to the CM, indicating SBCA’s high reactivity, facilitating the hydration process, and forming more densified CSH gel. Furthermore, the lowest Ca value was observed for the SCBA-P10. The decrease in Ca content is mainly due to the consumption of more Ca for the formation of CSH gel, which validates the increase in CS and split tensile strength.
- N_2_ adsorption results revealed that the addition of SCBA densifies the pore structure when used as a cement replacement. The decrease in induced pore volume and increased surface area indicate improved pore structures for all SCBA-based matrices compared to the CM. The lowest induced pore volume and highest surface area were noticed for the cement replacement at 10% of SCBA.
- XRD and SEM-EDX analyses confirm that SCBA undergoes a reactive silica-calcium hydroxide interaction, refining the microstructure and enhancing strength. N₂ adsorption analysis reveals reduced porosity and refinement of pore structure in SCBA-based mixes, resulting in crack resistance and durability. These microstructural improvements directly correlate with the observed mechanical strength gains, validating SCBA’s effectiveness as a supplementary cementitious material for stronger, more durable concrete.
- The cost analysis and carbon emissions results demonstrated that cost and CO_2_ emissions were reduced by increasing the amount of SCBA in the concrete. The findings suggest that the addition of 10% of SCBA (SCBA-P10) as a partial replacement of cement in concrete was more favorable in terms of strength, environment, and economy.

The feasibility of using SCBA as a partial replacement of cement for concrete revealed immense potential in terms of enhanced mechanical strength, reduction in CO_2_ emissions, and costs. However, further studies on the comprehensive understanding of the corrosion process in SCBA-based concrete, and the durability of SCBA-based concrete with distinct types of cement, admixtures, and various W/B ratios are recommended. Additionally, future studies should systematically evaluate the long-term durability performance of SCBA-based concrete through standardized testing of chloride penetration, carbonation depth, and cyclic wet-dry exposure. Furthermore, future researchers should adopt advanced mixing techniques like high shear mixing or ultrasonic dispersion for SCBA effective dispersion to overcome the problems encountered due to the mixing procedure. The potential for large-scale implementation of SCBA-modified concrete varies by region, showing particular suitability for areas with substantial sugarcane production (Brazil, India, Thailand) and strong environmental construction policies. The three major obstacles currently limiting broader application are inconsistent composition of raw ash materials, economically unfeasible preparation requirements, and insufficient recognition in construction regulations. Overcoming these limitations will require coordinated efforts between researchers, industry stakeholders, and policymakers to establish reliable material standards, develop efficient production techniques, and achieve formal code approval for this eco-friendly cement replacement.

## Data Availability

The datasets used and/or analyzed during the current study are available from the corresponding author on reasonable request.
